# Supplementary material for: The role of Nef in the long-term persistence of the replication-competent HIV reservoir in South African women
Source: J Virol. 2025 Jun 24;99(7):e00217-25. doi: 10.1128/jvi.00217-25 (PMC12282088; doi:10.1128/jvi.00217-25)
Supplement: Supplemental figures — Figures S1 to S7. [file jvi.00217-25-s0001.docx]

Supplementary Information:

The role of Nef in the long-term persistence of the replication-competent HIV reservoir in South African women

**Authors:**

Sherazaan D. Ismail^1*^, Shorok Sebaa^1^, Bianca Abrahams^1^, Martha C. Nason^2^, Mitchell J. Mumby^3^, Jimmy D. Dikeakos^3^, Sarah B. Joseph^4,5^, Matthew Moeser^5^, Ronald Swanstrom^4,5,6^, Nigel Garrett^7,8^, Carolyn Williamson^1,7,9^, Thomas C. Quinn^10^, Melissa-Rose Abrahams^1#^, Andrew D. Redd^1,10,11#^*.

Affiliations:

^1^Institute of Infectious Disease and Molecular Medicine, University of Cape Town, Cape Town, South Africa.

^2^Biostatistics Research Branch, Division of Clinical Research, National Institute of Allergy and Infectious Diseases, NIH, Bethesda, MD, USA.

^3^Department of Microbiology and Immunology, Schulich School of Medicine and Dentistry, Western University, London, ON, Canada.

^4^Department of Microbiology & Immunology; University of North Carolina at Chapel Hill, Chapel Hill, NC, USA.

^5^Lineberger Comprehensive Cancer Centre, University of North Carolina at Chapel Hill, Chapel Hill, NC, USA.

^6^Department of Biochemistry & Biophysics, University of North Carolina at Chapel Hill, Chapel Hill, NC, USA.

^7^Centre for the AIDS Programme of Research in South Africa, University of Kwazulu-Natal, Durban, South Africa.

^8^Department of Public Health Medicine, School of Nursing and Public Health, University of KwaZulu-Natal, Durban, South Africa.

^9^National Health Laboratory Services of South Africa, Johannesburg, South Africa.

^10^Division of Infectious Diseases, Department of Medicine, Johns Hopkins University School of Medicine, Baltimore, MD, USA.

^11^Laboratory of Immunoregulation, Division of Intramural Research, National Institute of Allergy and Infectious Diseases, NIH, Bethesda, MD, USA.

#-Shared senior authorship

*Corresponding authors: [reddandrew@niaid.nih.gov](mailto:reddandrew@niaid.nih.gov); [sherazaan.ismail@uct.ac.za](mailto:sherazaan.ismail@uct.ac.za)

**Supporting information captions and figures (figure after caption):**

**Figure S1. Individual participant Nef amino acid trees.** OGV *nef* sequences were translated, aligned by Clustal W in MEGA11 [1] and trees were generated using PhyML [2] in DIVEIN [3]. Trees were mid-point rooted and sorted by decreasing node order. A scale bar is included below each tree. Labels indicate the time pre-ART that each OGV was estimated to enter the reservoir in weeks (wks). Red node labels indicate amino acid sequences that were selected for synthesis and subsequent testing for function. Black node labels represent OGV sequences that were not selected for further functional testing in this study.


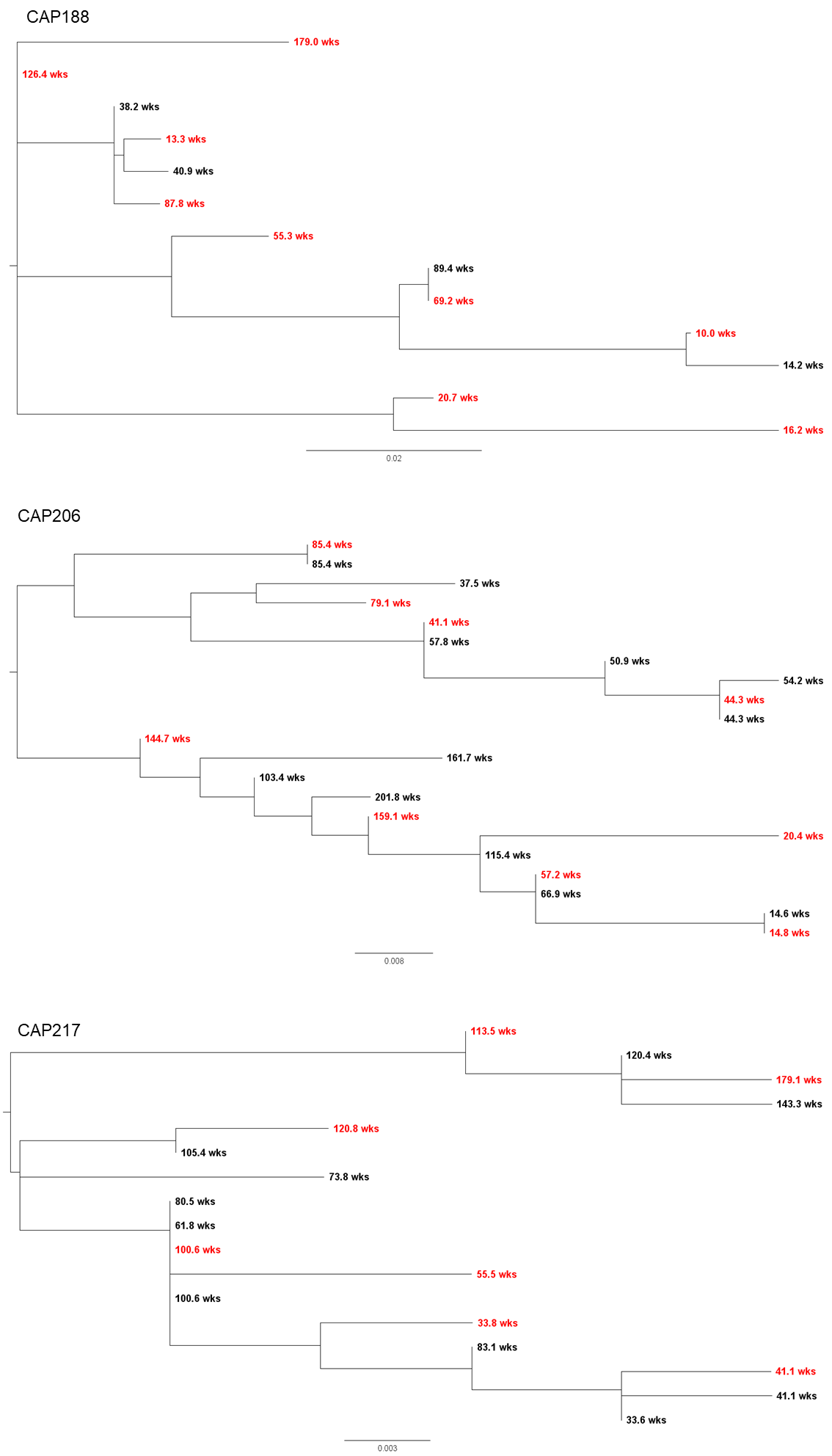


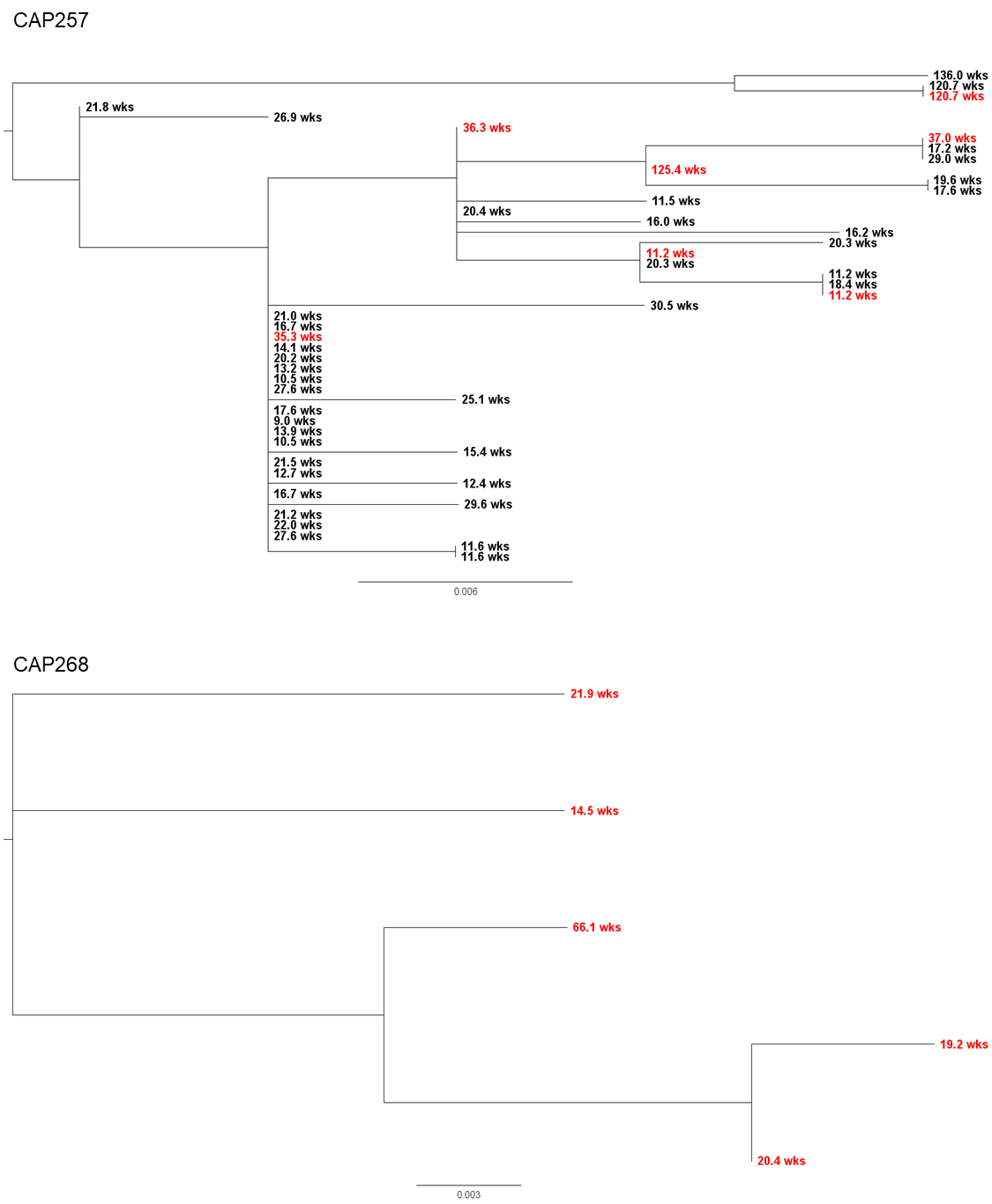


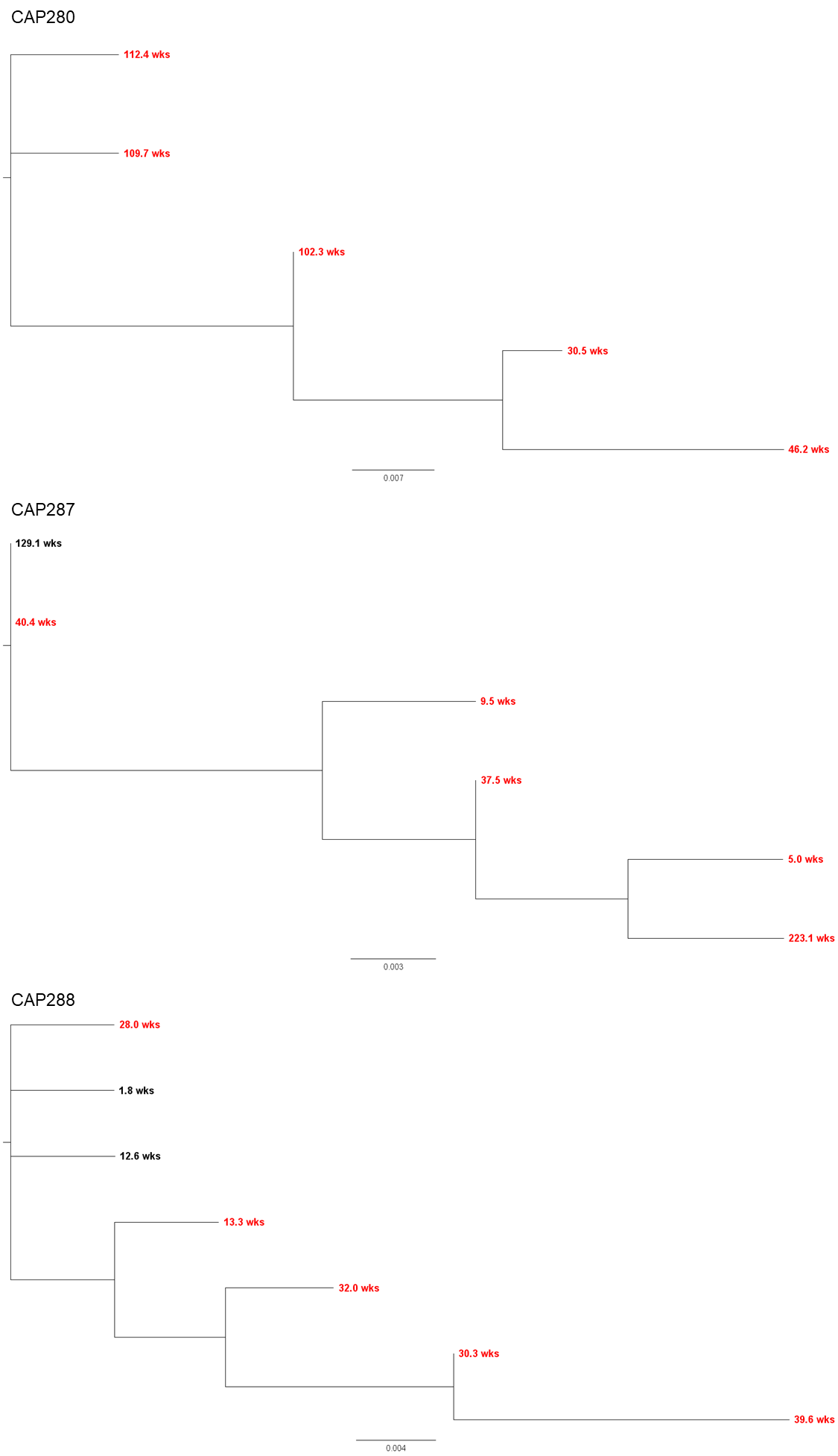


**
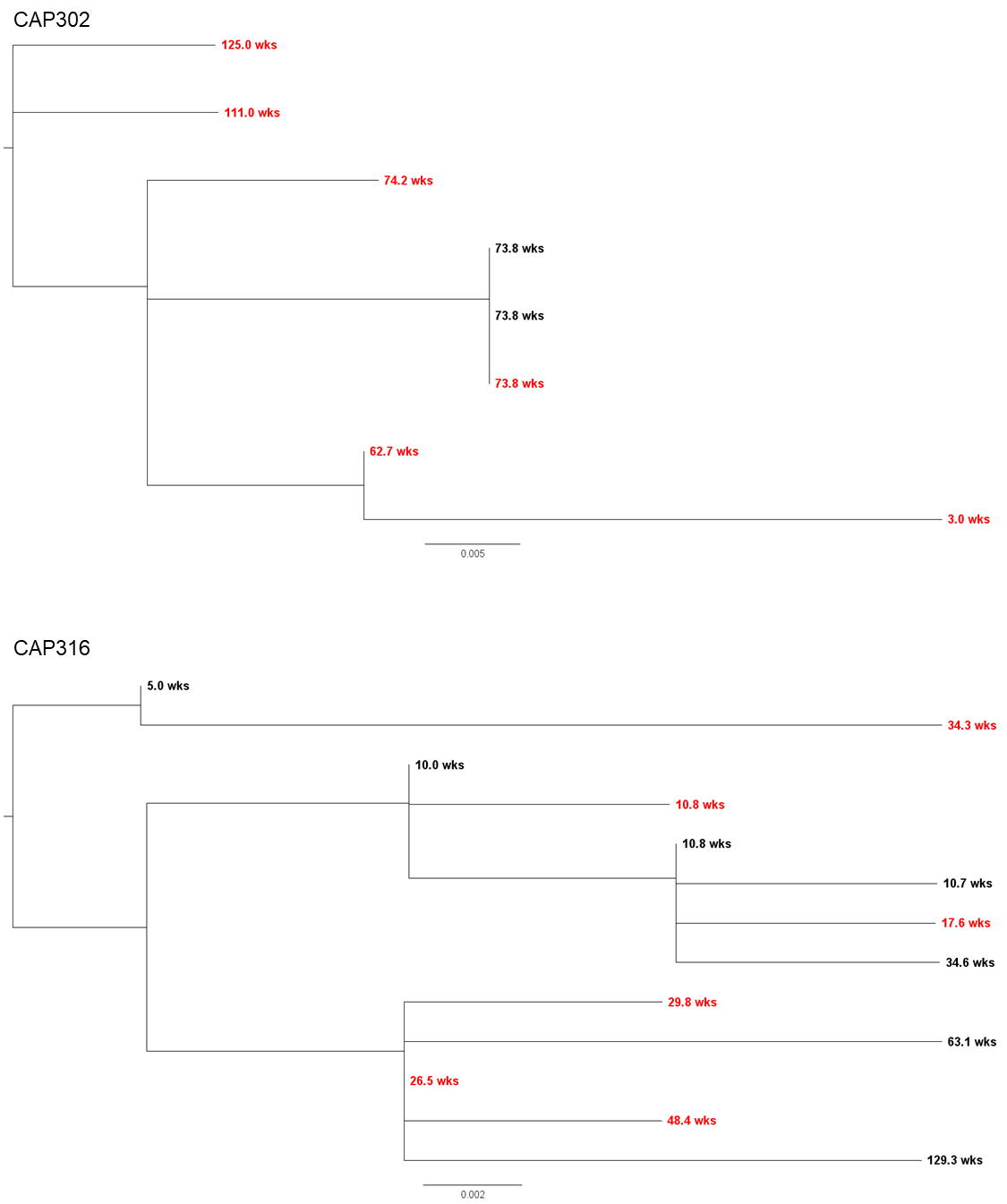
**

**
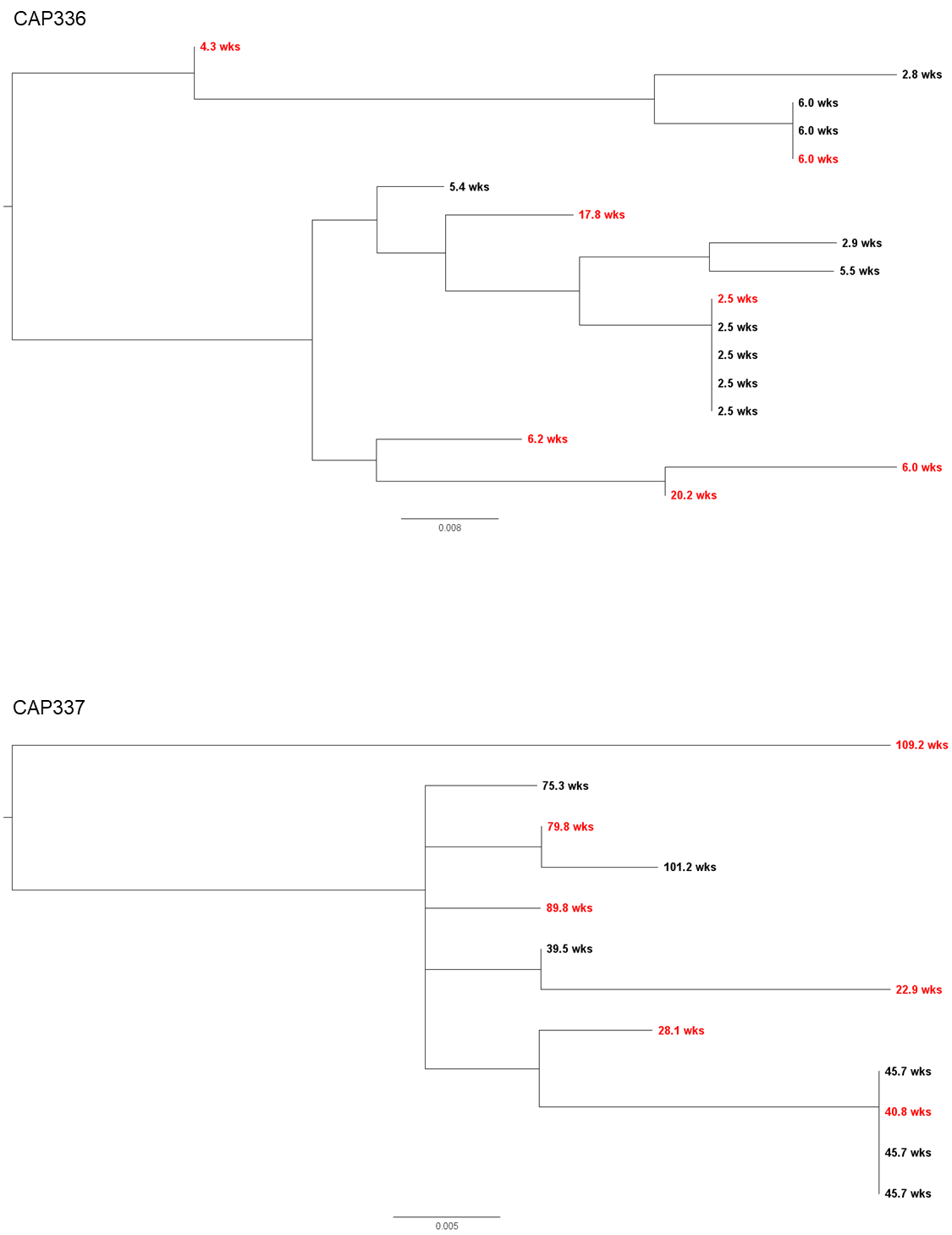
**

**Figure S2. Estimated timing of reservoir entry distribution of Nefs selected for functional analyses.** The timeline shows the estimated timing of entry into the reservoir relative to ART initiation (% of total untreated infection) of each selected outgrowth virus Nef. ART initiation is represented as x=0% and participant IDs are listed on the y-axis. Data points are coloured by participant.

**Figure S3. Gating strategy.** Live infected Sup-T1 cells were infected with Nef-pseudotyped viruses. Sup-T1 cells were identified, and doublets were excluded before gating for live cells (gates indicated in purple) which were Near-Infrared^-^ (viability dye). Subsequently, infected cells were gated as eGFP^+^ events (red gate) while uninfected cells were identified as eGFP^-^ (blue gate). In this representative plot, 28.5% of live, single cells were infected. Gates were set based on single stained Sup-T1 cells as well as fluorescence minus one controls (FMOs), stained for all **fluorophores except that which is being gated on. Histogram overlays for either CD4 or MHC-I** expression show the fluorescence intensity shift between infected cells (red peaks) and uninfected cells (blue peaks).

**
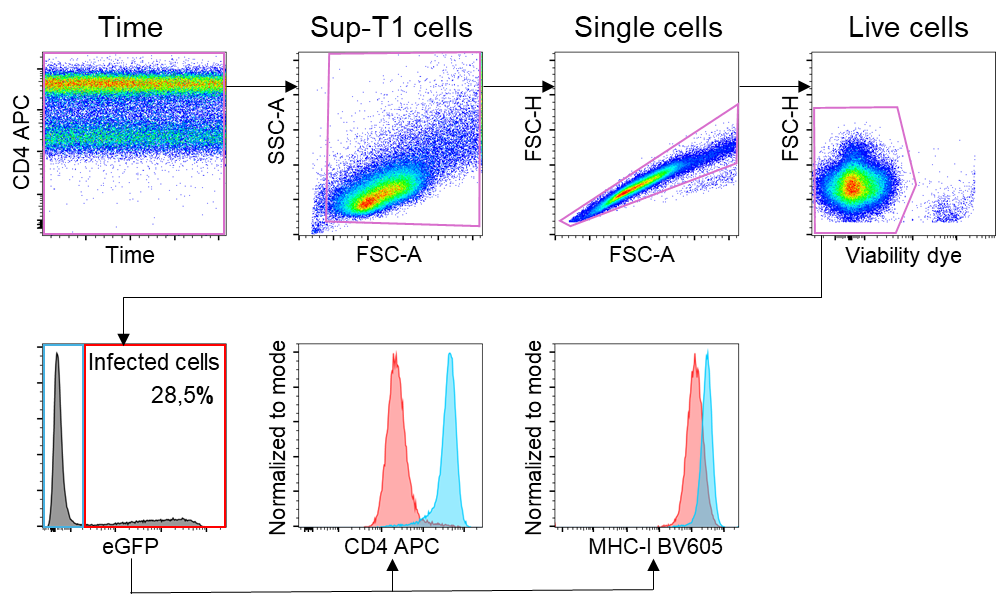
**

**Figure S4. Representative flow plots showing CD4 and MHC-I downregulation on the cell surface of infected (eGFP+) versus uninfected (eGFP-) cells from the same well.** Each row of figures shows flow plots for one well corresponding to the sample listed on the left of the figure. Blue peaks represent the uninfected population of cells (i.e. eGFP- cells) while red peaks represent the infected population of cells (i.e. eGFP+ cells) in each well. The ΔNef infection results from a pseudovirus produced using a plasmid encoding eGFP without the Nef (i.e. no fusion peptide present).

**
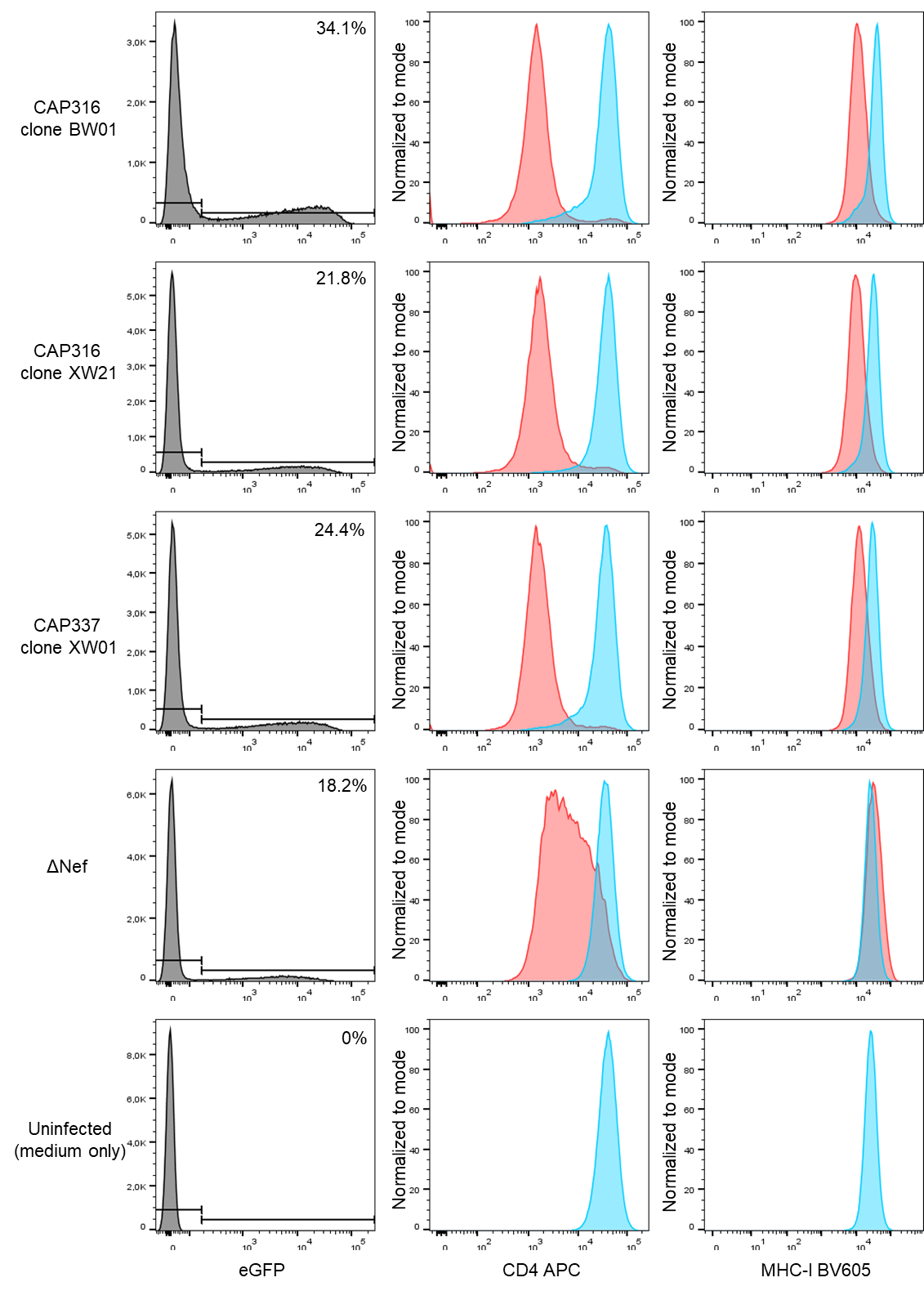
**

**Figure S5. Relationships between reservoir size, maximal MHC-I downregulation, or maximal CD4 downregulation and age, viral burden during untreated infection, or nadir CD4 count.** Each point on the graph represents a single value for a participant. Where a significant correlation exists between variables, a linear regression best fit line with 95% CI is plotted. P-values and best-fit line slopes are on the bottom right of their respective panels. Reservoir size is represented as the number of infectious units per million CD4^+^ T cells (IUPM). Viral loads were calculated using area under the curve (AUC VL expressed as time.copies/mL) from 3 months post estimated date of infection until ART initiation, excluding peak viraemia.

**
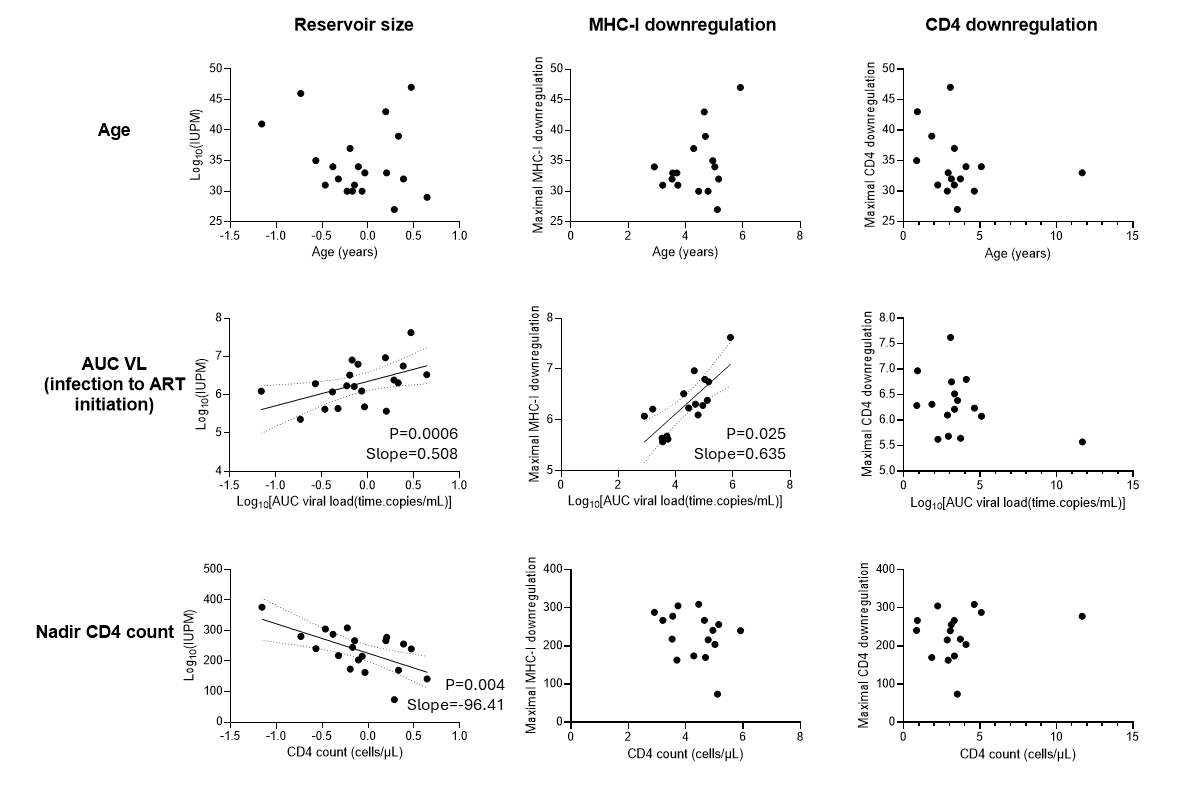
**

**Figure S6.** **Participants with a significant linear relationship between MHC-I downregulation activity and proviral survival time.** Each point on the graph represents geometric mean MHC-I downregulation for a unique *nef* clone and the error bars represent the 95% CI. Linear regression best fit lines and 95% CI were plotted for significant linear relationships.


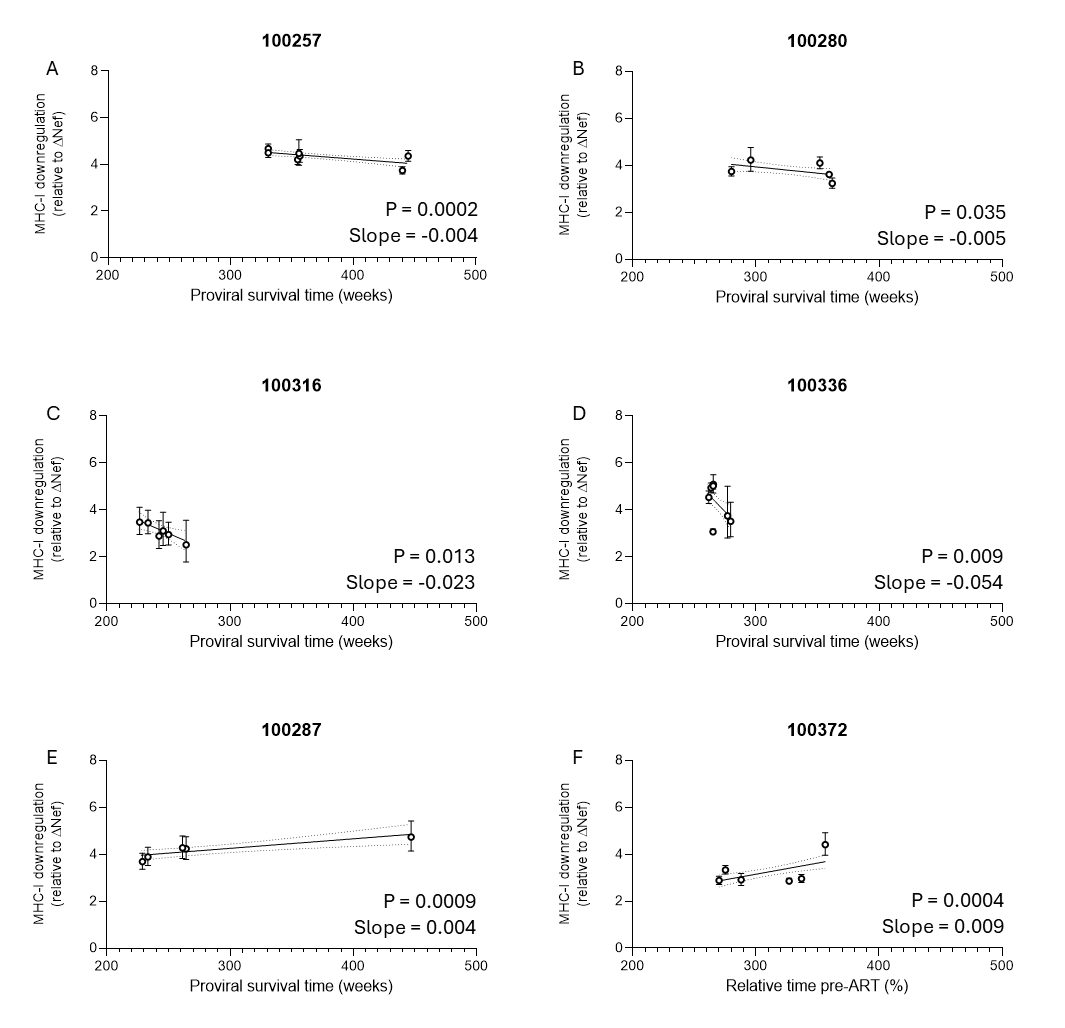


**Figure S7. Individual participants with a significant linear relationship between CD4 downregulation activity and proviral survival time.** Each point on the graph represents geometric mean CD4 downregulation for each *nef* clone and the error bars represent the 95% CI. Linear regression best fit lines and 95% CI were plotted for significant linear relationships.


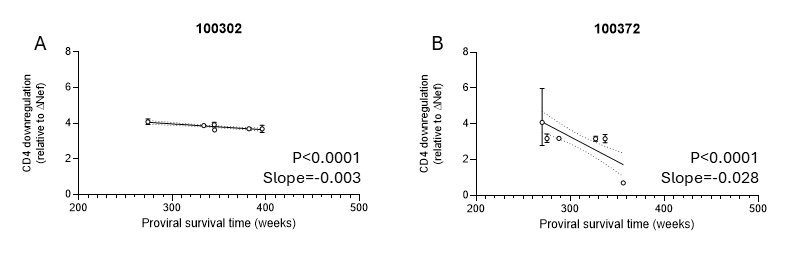


**References:**

1. Tamura K, Stecher G, Kumar S. MEGA11: Molecular Evolutionary Genetics Analysis Version 11. Battistuzzi FU, editor. Mol Biol Evol. 2021;38: 3022–3027. doi:10.1093/molbev/msab120

2. Guindon S, Dufayard JF, Lefort V, Anisimova M, Hordijk W, Gascuel O. New algorithms and methods to estimate maximum-likelihood phylogenies: Assessing the performance of PhyML 3.0. Syst Biol. 2010;59: 307–321. doi:10.1093/sysbio/syq010

3. Deng W, Maust BS, Nickle DC, Learn GH, Liu Y, Heath L, et al. DIVEIN: a web server to analyze phylogenies, sequence divergence, diversity, and informative sites. Biotechniques. 2010;48: 405–408. doi:10.2144/000113370
